# Call for new criteria for monitoring and registering Natura 2000 species data

**DOI:** 10.1111/cobi.70064

**Published:** 2025-05-31

**Authors:** Meritxell Genovart, Roberto Salguero‐Gomez, Fernando Colchero, Francisco Guil, Joan Rabassa‐Juvanteny, Julia Uriach‐Dasca, Dalia Amor Conde, Jean Michel Gaillard, Tim Coulson

**Affiliations:** ^1^ Department of Ecology and Complexity CEAB (CSIC) Blanes Spain; ^2^ Department of Biology University of Oxford Oxford UK; ^3^ Department of Primate Behavior and Evolution Max Planck Institute for Evolutionary Anthropology Leipzig Germany; ^4^ Department of Mathematics and Computer Science University of Southern Denmark Odense Denmark; ^5^ Spanish Ministry for the Ecological Transition and the Demographic Challenge Madrid Spain; ^6^ Department of Biology University of Southern Denmark Odense Denmark; ^7^ Unité de Recherche Mixte 5558 «Biométrie et Biologie Evolutive» Université Lyon 1 Villeurbanne France

**Keywords:** biodiversity, conservation biology, conservation policy, life‐history strategies, management, monitoring, Natura 2000, biodiversidad, biología de la conservación, estrategias de historias de vida, gestión, monitoreo, Natura 2000, políticas de conservación

## Abstract

The European Union's Birds and Habitats Directives are intended to guarantee the persistence of species and natural habitats across member states. To achieve this laudable aim, the Natura 2000 network of protected areas was established in 1992. Since then, member states are required to regularly monitor species and habitats and report findings to the European Commission, which requires substantial investment from all countries. The Natura 2000 network is an invaluable example of a large‐scale coordinated network developed to address major conservation issues. Based on our analysis of the 2020 Species Natura 2000 database and on expert opinions by Natura 2000 executives, we found that the network is failing to adequately show biodiversity status and guide conservation because it does not allow cross‐border comparisons of species’ and populations’ conservation status. The main contributing factor to this failure is that member states frequently fail to follow reporting EU guidelines, resulting in heterogeneity in criteria for monitoring and registering species among Natura 2000 areas. We advocate developing new unified and realistic criteria for monitoring and reporting species data that correctly allow cross‐border comparisons and conservation diagnosis. We propose that monitoring protocols and current criteria be modified slightly by considering species’ life‐history strategies, distribution, and conservation status. We do not suggest a major overhaul of the directives; rather, we propose debate on how relatively small changes in guidelines could improve the utility of the huge amount of data collected from the Natura 2000 network.

## Abstract (es)

Nuevos criterios para el monitoreo y registro de especies en Natura 2000

**Resumen:**

Las Directivas de Aves y Hábitats de la Unión Europea pretenden garantizar la persistencia de especies y hábitats naturales en todos los Estados miembros. Para lograr este loable objetivo, en 1992 se creó la red de áreas protegidas Natura 2000. Desde entonces, se exige a los Estados miembros que controlen periódicamente las especies y los hábitats y comuniquen los resultados a la Comisión Europea, lo que requiere una inversión considerable por parte de todos los países. La red Natura 2000 es un ejemplo invaluable de una red coordinada a gran escala desarrollada para abordar grandes problemas de conservación. A partir de nuestro análisis de la Base de Datos Natura 2000 de Especies 2020 y de las opiniones de expertos de los directivos de Natura 2000, descubrimos que la red no muestra adecuadamente el estado de la biodiversidad ni orienta la conservación porque no permite hacer comparaciones transfronterizas del estado de conservación de especies y poblaciones. El principal factor que contribuye a este fracaso es que, con frecuencia, los Estados miembros no siguen las directrices de la UE en materia de presentación de informes, lo que da lugar a una heterogeneidad en los criterios de seguimiento y registro de especies entre las áreas Natura 2000. Recomendamos desarrollar nuevos criterios unificados y realistas de seguimiento y notificación de datos sobre especies que permitan que las comparaciones transfronterizas y el diagnóstico de conservación sean correctas. Proponemos que los protocolos de seguimiento y los criterios actuales se modifiquen ligeramente teniendo en cuenta las estrategias de vida, la distribución y el estado de conservación de las especies. No sugerimos una revisión a fondo de las directrices, sino que proponemos un debate sobre cómo unos cambios relativamente pequeños podrían mejorar la utilidad de la enorme cantidad de datos recogidos en la red Natura 2000.

## INTRODUCTION

Natura 2000 is a European Union (EU) network of protected areas established to implement the Birds Directive (Directive 2009/147/EC) and the Habitats Directive (Council Directive 92/43/EEC), collectively known as EU nature legislation and intended to guarantee the persistence of species and natural habitats across EU Member States. Natura 2000 is a prime example of a large‐scale coordinated network developed and operated to address major conservation issues across international borders and constitutes a fundamental tool for the conservation of European biodiversity. The network was established in 1992, and, at present, it covers nearly 27,000 sites and protects more than 1.2 million km^2^, 18% of the EU land area and almost 10% of its marine territory (European Commission, 2020; IEEP and the N2K Group, 2022). It is intended to improve and widen Europe's network of protected areas so that at least 30% of the land and 30% of the sea is protected (IEEP and the N2K Group, 2022). Managing the Natura 2000 network requires substantial investments. The best estimate of financing needs for the 27 EU Member States is €10.6 billion annually.

Since the establishment of the network, European Member States have been required to monitor selected species and habitats outside and inside the network and to assess their conservation status. Species to be monitored are those included in the Birds Directive Annexes I and II (Council Directive 2009/147/EC) and in the Habitats Directive Annexes II, IV, and V (Council Directive 92/43/EEC) and regularly migratory species (e.g., Greylag goose [*Anser anser*] and Gadwall [*Mareca strepera*]). Monitoring data from inside the network are reported annually through the Natura 2000 Standard Data Form (SDF), the format for the transmission of information on the Natura 2000 network. The conservation status of species and habitats is reported every 6 years. These monitoring data are stored in a freely accessible database that is updated annually as new data are reported (https://sdi.eea.europa.eu/data/b1777027‐6c85‐4d19‐bdf2‐5840184d6e13).

In 2021, Natura 2000 managers contacted some of us to share their strong concerns about current criteria used to complete the SDF and asked for help in assessing whether their concerns were well founded and, if so, what the consequences were. Specifically, they considered that the highly restrictive criterion to determine the “significance of populations” (defined below) in the “site assessment” (Section 3.1.b of the SDF; Appendix S1) could lead to data heterogeneities among countries and even among regions within the same country. To assess the relevance of their concerns, we examined the quality and availability of the data gathered across the network and identified differences among EU countries; determined whether different member states used the same criterion to determine the significance of populations; and evaluated the possible consequences of disparities in criteria among member states.

## NATURA 2000 SPECIES DATA

An ongoing debate exists regarding the Natura 2000 network effectiveness at maintaining viable populations and how to improve it (Engelhardt et al., 2023; Gameiro et al., 2020; Gruber et al., 2012; Hermoso et al., 2019, 2018; Hochkirch et al., 2013; Kreft & Güngöroglu, 2018; Kukkala et al., 2016; Maes et al., 2013; Maiorano et al., 2007; Opermanis et al., 2013; Princé et al., 2021; Trochet & Schmeller, 2013). Some previous studies point out that sometimes data quality and availability appear to be a limitation (Campagnaro et al., 2019; Lisón et al., 2017; Zisenis, 2017). Of special interest here is the utility of monitoring data gathered by Natura 2000 for spatiotemporal analyses or comparison of the conservation status of populations and species.

**FIGURE 1:**
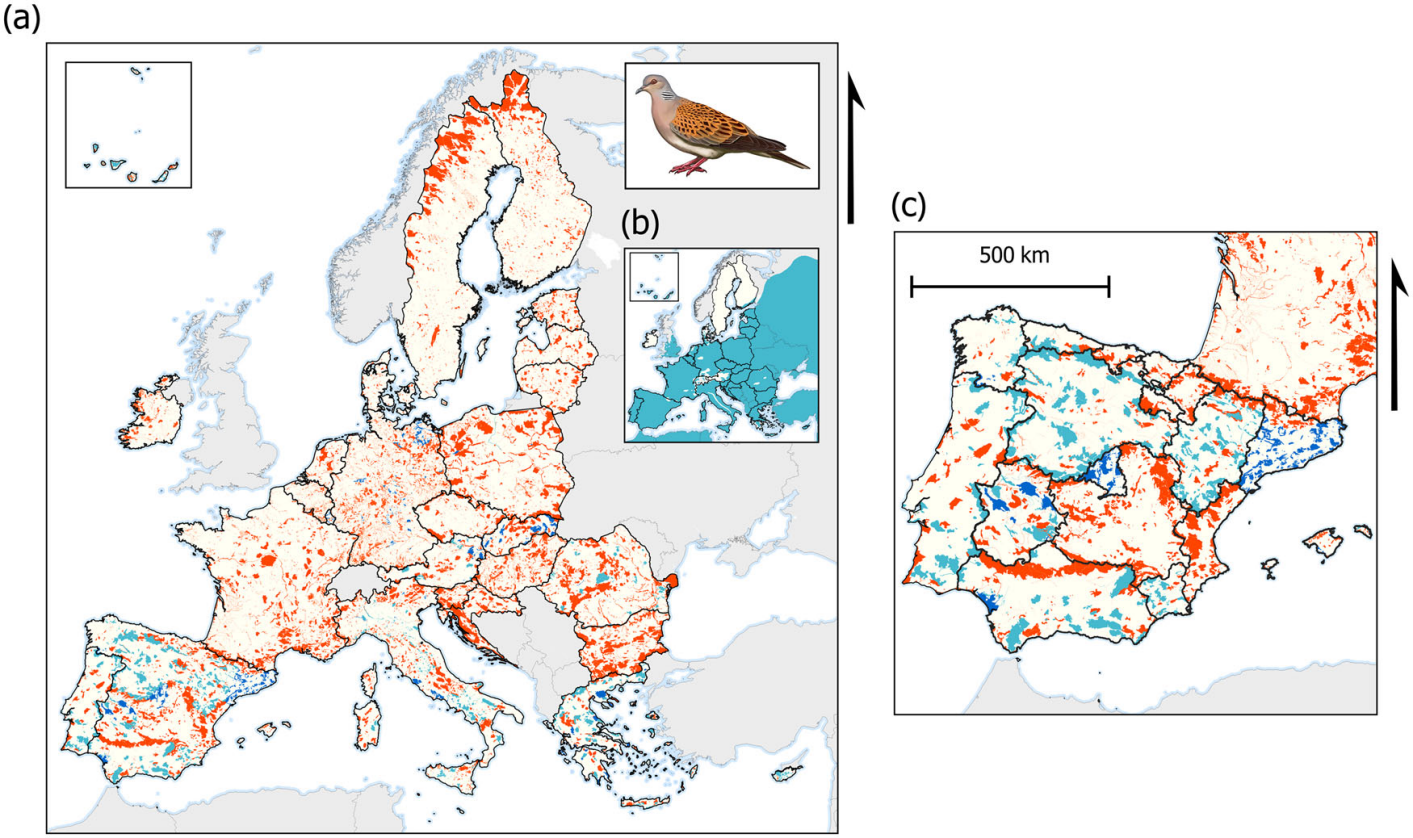
Distribution of the European turtle dove (*Streptopelia turtur*): (a) occurrence and population size from the 2020 Natura 2000 database, (b) distribution of the species based on data from BirdLife International (2022), and (c) occurrence and population size from the 2020 Natura 2000 database in Spain (light blue, 2000 Natura sites with registered presence of the species but no information on population size; dark blue, 2000 Natura sites with registered presence of the species and information on population size; red, 2000 Natura sites with no registered presence of the species; in [c], black lines, regional frontiers).

Differences in the availability and quality of the data among countries may compromise its utility. Section 3 of the SDF includes all the information of the species listed in the directives in an area. In the population section of the form, data quality has 4 levels: good (e.g., complete survey or a statistically robust estimate); moderate (e.g., based on partial data with some extrapolation); poor (e.g. rough estimation); and data deficient (when not even a rough estimation can be made). We analyzed the 2020 Species Natura 2000 database and specifically evaluated the availability (i.e., missing data) and quality (i.e., typological and completion errors in the data and data quality level of population abundance section) of the data across the network (detailed description of the SDF in Appendix S1). We detected that data contained typological and completion errors, and information on multiple species was entirely lacking or incomplete (e.g., >50% of species were missing population size data) (Appendix S1); there was heterogeneity in the data across member states (Figure 1; Appendix S1); and even substantial variation existed among regions within countries (Figure 1c; Appendix S1). These differences among states or regions were evident in terms of the quantity and quality of the data reported in different sections of the SDF and in the monitoring effort for different taxonomic groups (Appendix S1).

## DEFINITION OF *SIGNIFICANT POPULATIONS*

Some of the definitions in the SDF have been criticized. For instance, among Natura 2000 managers, there is general concern about the definition of *significant populations* when assessing the population category in the SDF. Given that *p* is the size of the population in the area in relation to populations present within the national territory, populations should be classified as significant if 100% ≥ *p* > 15%, 15% ≥ *p* > 2%, or 2% ≥ *p* > 0%, corresponding to categories A, B, and C, respectively. Thus, the European Commission recommends that only those species “rarely” observable at the site, for example, a vagrant species, should be officially accepted as “not significant,” category D.

In cases where the population is classified as not significant, no other indication is required for the other evaluation criteria concerning this species on the site in question. This criterion is critical because conservation objectives and effective measures have to be set or referred to in legally binding acts for all species with “significant” presence in each Natura 2000 area (https://eur‐lex.europa.eu/legal‐content/EN/TXT/PDF/?uri=CELEX:32011D0484), but they do not need to be set for “nonsignificant” populations. Given that resources devoted to conservation are limited and biodiversity is not uniformly distributed among countries, this very strict criterion may be an additional factor favoring the occurrence of arbitrary decisions for the allocation of resources among countries and regions.

By directly consulting with the Biodiversity Natura 2000 managers in each country, we confirmed that expert opinion is widely used by many state members for some or all the decision‐making steps in the assessment of Natura 2000 populations, especially to determine the significance of populations (Appendix S2). For instance, Spain and Germany, even if they officially use the recommended European commission criterion, in practice are using expert opinion to determine the significance of populations (Appendix S2). Another example is the Netherlands, which explicitly uses a particular quantitative criterion that is not as restrictive as the one recommended by Natura 2000 (Appendix S2).

## CONSEQUENCES OF CRITERIA HETEROGENEITIES AMONG COUNTRIES

In assessments of the distribution and availability of population size data on several species based on recorded data on Natura 2000, we found that the consequences of the disparities across member states in effort and allocation of resources and in criteria used to identify significant populations were not negligible. Indeed, the aforementioned disparities generated great inconsistencies in species perceived distribution among countries, likely consequences of territorial limits instead of true species distributions (Figure 1; Appendix S3). For instance, based on Natura 2000 data, the common European turtle dove (*Streptopelia turtur*) shows an unexpected distribution across Europe that seems mostly unrepresentative of the species actual distribution, including sharp border limits at the national and regional levels (Figure 1) (BirdLife International, 2022).

Although our approach does not provide a rigorous comparison of the maps of the species distribution—because such a comparison would require overlaying high‐resolution occurrence data in the Natura 2000 database with those in other more comprehensive databases—it does highlight problems associated with current data monitoring protocols. To more accurately assess the accuracy of the Natura 2000 network data, future research should extend our visual analysis with advanced quantitative methods. However, our assessment did show, in many species, there were marked differences in monitoring effort. Some countries or regions had no data, some monitored population size, and others indicated only presence or absence of the species at a given location (Figure 1; Appendices S1 & S3). We found that these heterogeneities resulted in a data set that does not allow for the objective assessment or comparison of the conservation status of species across Europe.

## CALL FOR NEW CRITERIA

The aims of the Birds and Habitats Directives to preserve biodiversity are important and widely supported (Campagnaro et al., 2019). Much of the data collected across the Natura 2000 network sites are of high quality and potentially useful. However, the overall utility of the species 2000 network database is compromised by data errors and especially by lack of data comparability across regions and countries. This lack of consistency in data quality is partially due to the demands that the directives impose on those who collect data. Specifically, the highly restrictive criterion to determine the significance of populations implies the need to annually monitor large numbers of species, particularly in habitats with a high biodiversity. We argue that the use of different criteria across countries and regions and variation in the use of expert opinion are sources of heterogeneity in monitored species and populations and that this heterogeneity hinders the potential of the network. Data errors and lack of comparability could be corrected by improving the entire work flow, from data collection to data storage, through common data gathering and management procedures. This would include the implementation of mechanisms and tools to ensure data quality at several steps in the process.

**FIGURE 2:**
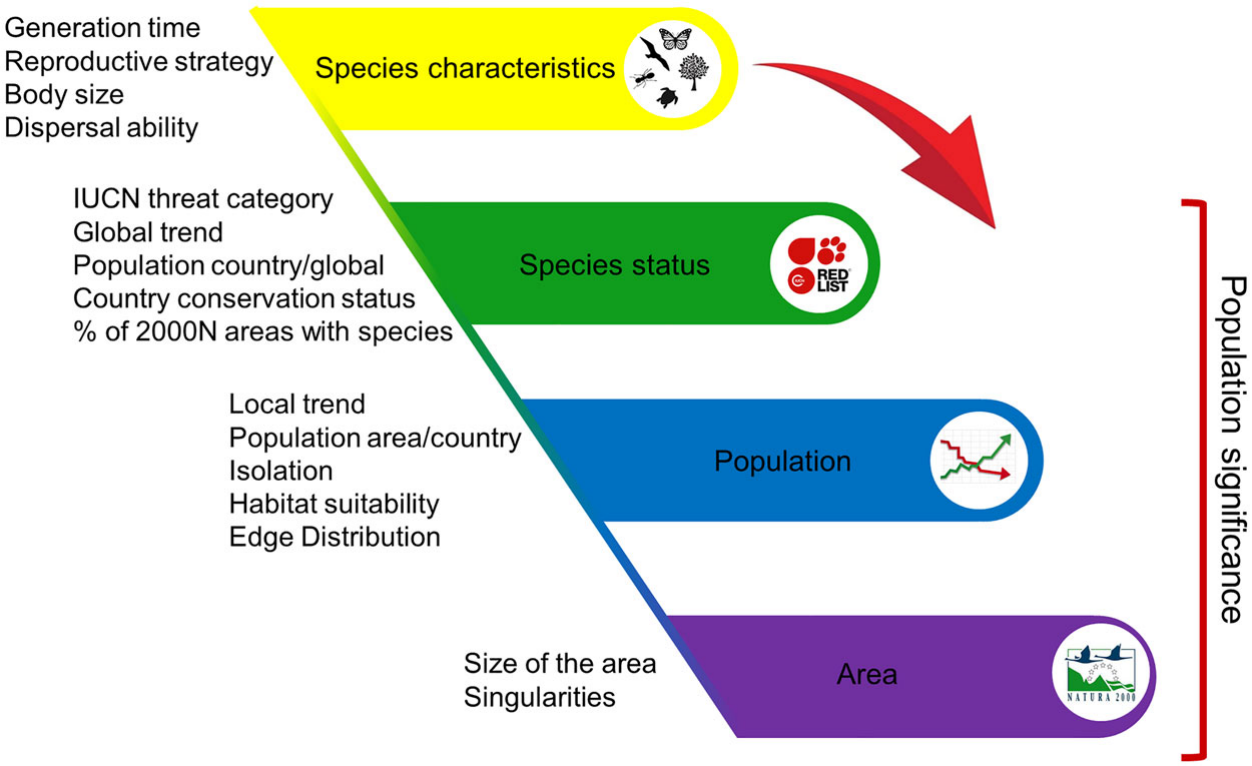
Species, population, and site characteristics to consider when defining monitoring protocols and determining population significance criteria.

We thus suggest 2 main modifications, one related to monitoring protocols and the other related to the definition of *significant populations*. For monitoring protocols, first and foremost, it is essential to report not only data but also metadata on monitoring efforts and monitoring protocols for each species and each Natura 2000 area. Some efforts in this direction can be already seen in the new proposed SDF (operative from 1 February 2025; European Environment Agency, 2025). In the new SDF, instead of just categorizing the quality of the population data, respondents are required to indicate the method used for assessing population size (i.e., “complete survey or a statistically robust estimate,” assessment “based mainly on expert opinion with very limited data,” assessment “based mainly on extrapolation from a limited amount of data,” or “insufficient or no data available”). Although this is a step in the right direction, providing more information is crucial for accounting for potential differences among countries in assessing Natura 2000 species, as well as for accurately comparing species population trends and distributions with data from other sources. Providing monitoring efforts and monitoring protocols for each species and each Natura 2000 area would radically increase the potential of this database (Bowler et al., 2021; Isaac et al., 2020; Menger et al., 2024; Outhwaite et al., 2020). Second, we aimed here to trigger a scientific debate that could lead to some formal additional guidance that would narrow disparities when defining monitoring protocol efforts across countries and regions and vary as a function of species’ characteristics, such as generation time, and species’ conservation status and distribution (Figure 2).

**FIGURE 3:**
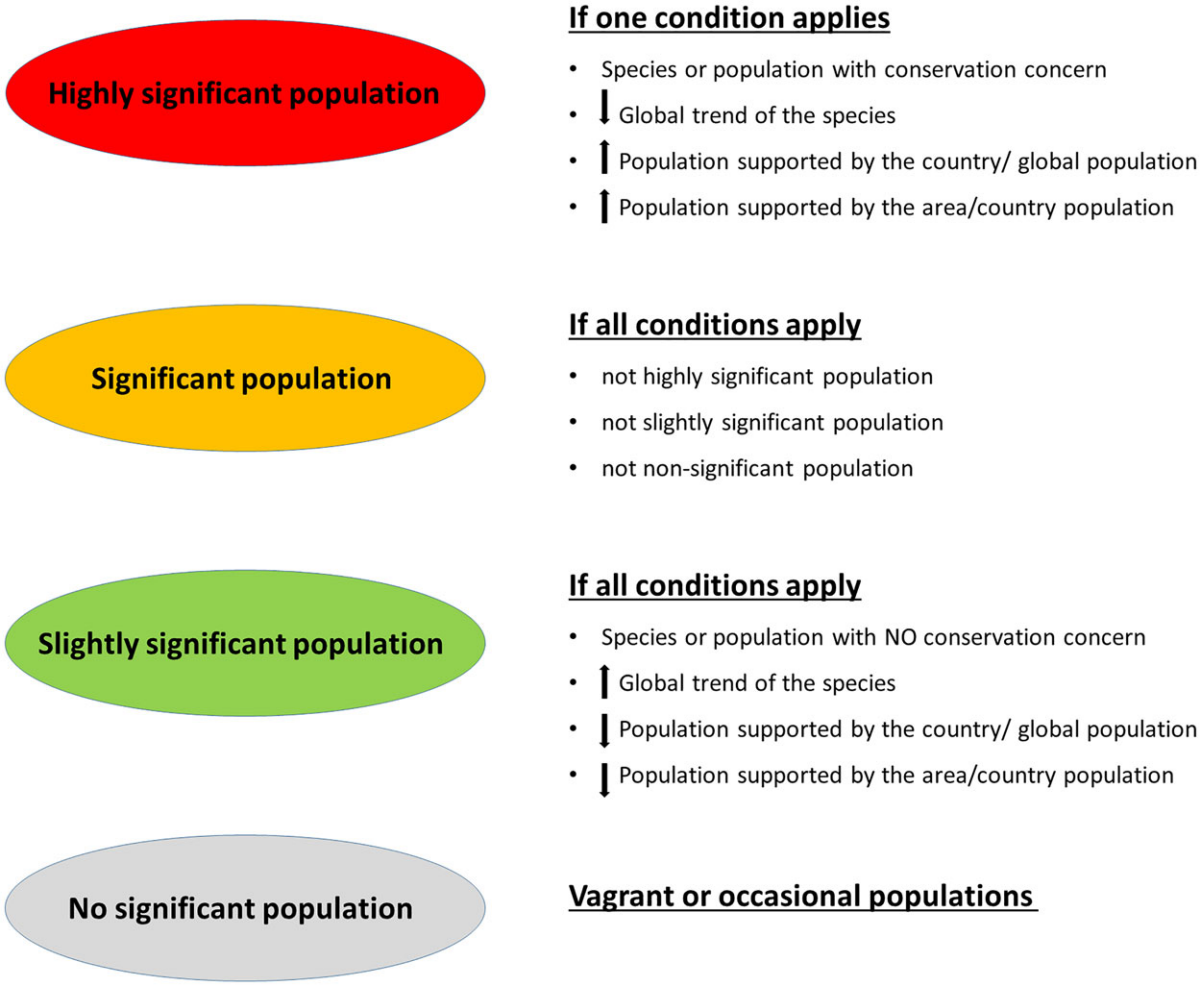
Possible subdivision of the significant population category to prioritize resources in a unified manner among European Union State Members.

We advocate too for a revised definition of *significant population*. One specific proposal would be to divide the current category significant population into 3 subcategories, each one entailing certain levels of prioritization in the allocation of resources and monitoring effort (Figure 3). These new criteria should consider the species’ trend, conservation status, and distribution at local and global scales and, in some cases, the characteristics of the area, such as the value of the site for conservation of the species concerned (Figures 2 & 3).

Moreover, the species‐specific life history should be considered when designing monitoring protocols and when determining species’ and populations’ conservation status in the SDF (Figure 2). Population trends of species with different life‐history strategies (i.e., different schedules of survival, development, and reproduction in a species’ life cycle) cannot be assessed in the same way and on the same time scale. Additionally, life‐history strategies determine the fragility, capacity to compensate, and finally the resilience of the species or populations facing different environmental changes (Capdevila et al., 2022; Dalgleish et al., 2010; Koons et al., 2016; Le Coeur et al., 2022; Morris et al., 2008).

We thus propose to enrich data dumping and slightly modify monitoring protocols and current criteria to determine population significance by considering species’ life‐history strategies, distribution, and conservation status (Figures 2 & 3). We are not suggesting a major overhaul of the directives but are instead proposing debate on how relatively small changes in guidelines could improve the utility of the huge amount of data collected from the Natura 2000 network. Biodiversity continues to decline globally, and the Natura 2000 network provides an invaluable resource in which to monitor and preserve the EU's biodiversity, but current practices could be markedly improved with relatively small tweaks to existing protocols and criteria.

## AUTHOR CONTRIBUTIONS

*Conceptualization*: Meritxell Genovart, Tim Coulson, Roberto Salguero‐Gomez, Fernando Colchero, and Francisco Guil. *Formal analyses*: Joan Rabassa‐Juvanteny and Meritxell Genovart. *Investigation*: Julia Uriach‐Dasca, Meritxell Genovart, Francisco Guil, and Joan Rabassa‐Juvanteny. *Funding acquisition*: Meritxell Genovart and Francisco Guil. *Writing original draft*: Meritxell Genovart and Tim Coulson. *Writing, review, and editing*: All authors.

## Supporting information



Supporting Information
