# Supplementary material for: Call for new criteria for monitoring and registering Natura 2000 species data
Source: Conserv Biol. 2025 May 31;39(5):e70064. doi: 10.1111/cobi.70064 (PMC12451482; doi:10.1111/cobi.70064)
Supplement: Supplementary file 1 — Supporting Information [file COBI-39-e70064-s001.docx]

Supporting Information for

Call for new criteria for monitoring and registering 2000 Natura species data

**The PDF file includes:**

- Supplementary Text, divided in 3 Appendices (1-3):
  - Appendix S1. Description of the Natura 2000 SDF and the SPECIES database
  - Appendix S2. Actual criteria to determine the “significance of populations”
  - Appendix S3. Analysis of species occurrence and data on population size

In the Supplementary Materials we first introduce the Natura 2000 SDF and provide a description of the relevant sections of the SDF and of the SPECIES database (Appendix 1); then we provide a detailed description of the actual criteria used to determine the “significance of populations” by different State Members (Appendix 2); and in the third appendix we show distribution maps of several species based on Nature 2000 database (Appendix 3).

The data used for this study belong to the European Environment Agency (https://ec.europa.eu/environment/nature/natura2000/data). Specifically, we used:

- The vector layer Natura 2000 End 2020 – Shapefile (year 2020)

- The Natura 2000 database - Tabular data - SPECIES (year 2020)

All the analyses were done in software R (R Core Team 2021; https://www.r-project.org/) and QGIS (https://www.qgis.org/es/site/).

**Appendix S1**. Description of the Natura 2000 SDF and the SPECIES database

Description of the section 3.2 of the Natura 2000 SDF

Natura 2000 is the ecological network for the conservation of wild animal and plant species and natural habitats of Community importance within the Union. It consists of the sites classified under the Birds Directive first adopted in 1979 (Directive 2009/147/EC) and the Habitats Directive adopted in 1992 (Directive 92/43/EEC). Natura 2000 SPECIES database is generated from the compilation of the SPECIES data annually reported from all countries in the Standard Data Form (SDF) (<http://eur-lex.europa.eu/LexUriServ/LexUriServ.do?uri=CELEX:32011D0484:EN:NOT>). See the Official Journal of the European Union (<https://eur-lex.europa.eu/legal-content/EN/TXT/PDF/?uri=CELEX:32011D0484>) for details on the SDF and instructions to fill information.

Section 3 of the SDF includes all the ecological information of a space. In section 3.1 habitats are reported, in section 3.2 species with reference to the Directives, and in section 3.3 other species not present in the Directives.

Specifically, article 3.2 is entitled 'Species to which article 4 of the Directive applies 2009/147/CE and species listed in Annex II of Directive 92/43/EEC and site evaluation depending on where you are and applies to ZEPAs, pLIC, LIC and ZEC. Section 3.2 of the SDF is shown below; first we show the SDF used until January 2025 and then the new one operative since 1^st^ February 2025.

Section 3.2 of the SDF operative until January 2025


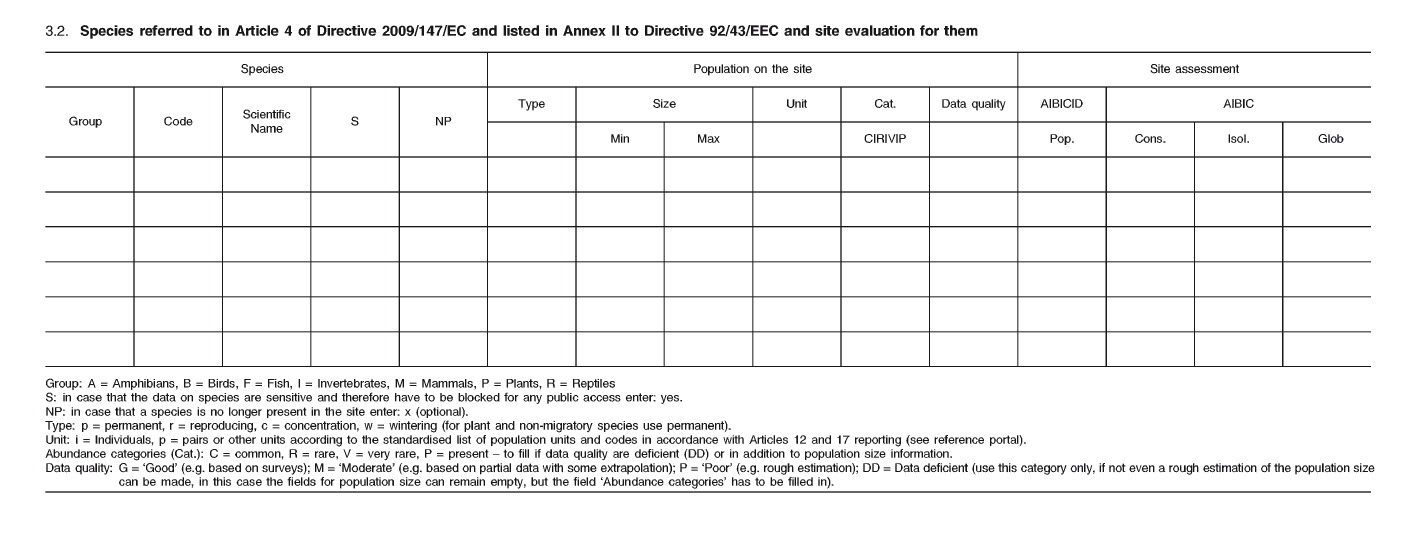


Section 3.2 of the SDF operative from 1^st^ January 2025


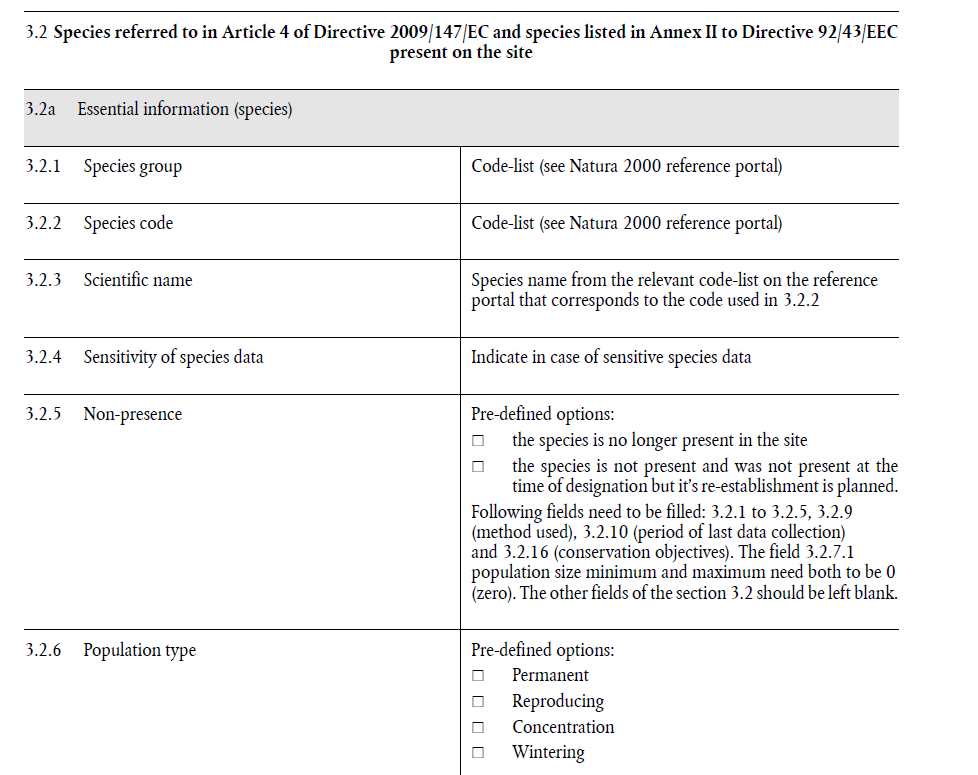

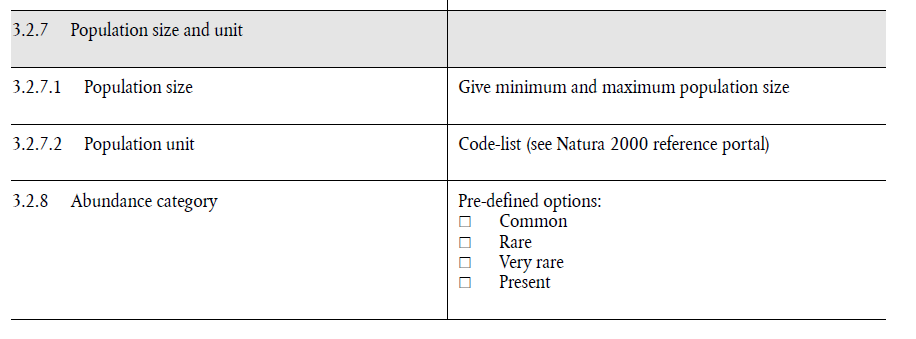

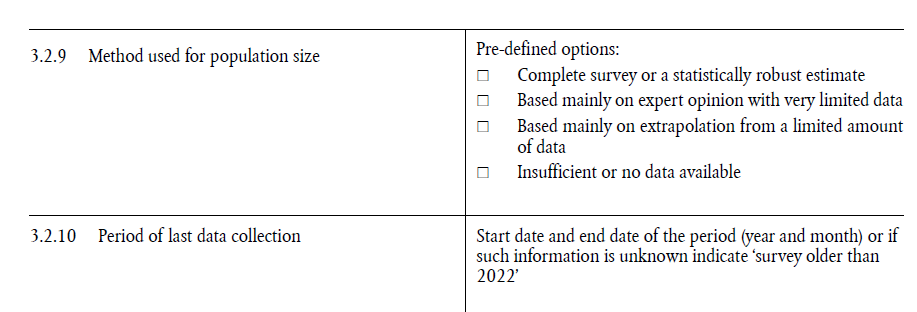

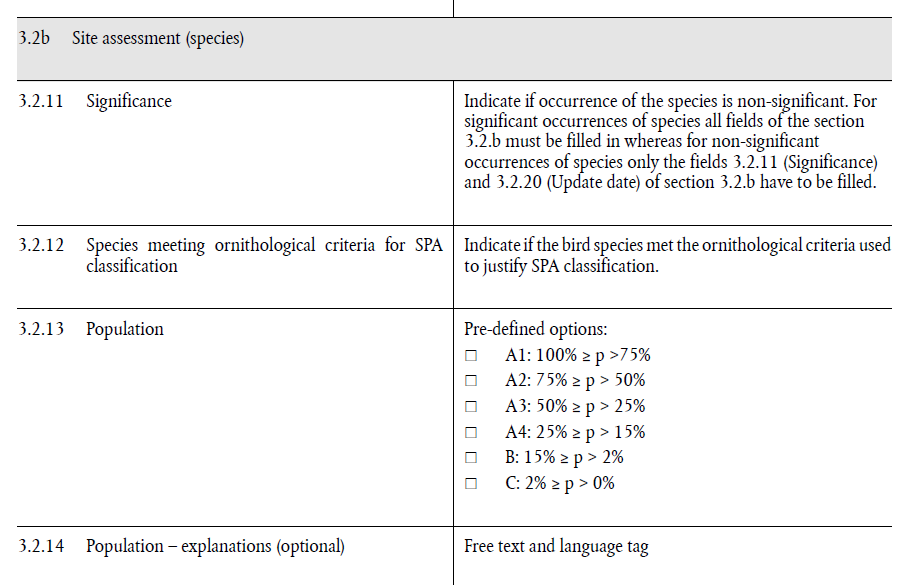

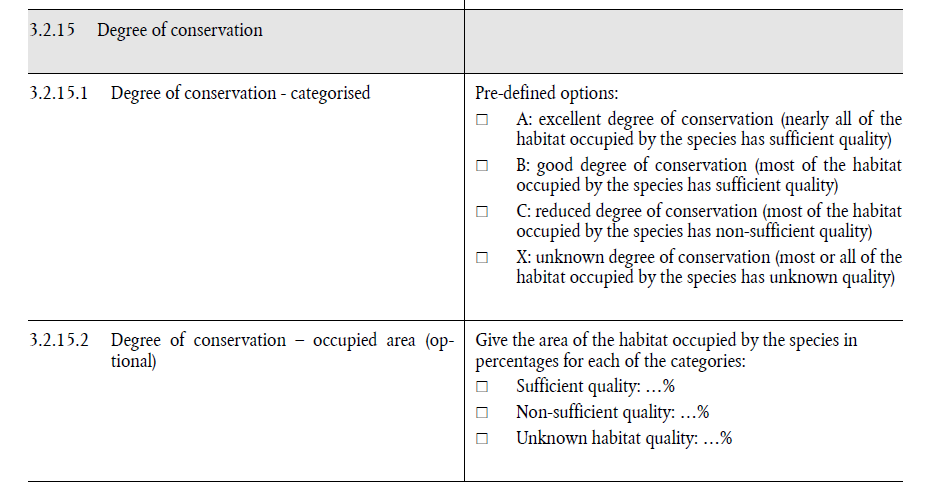

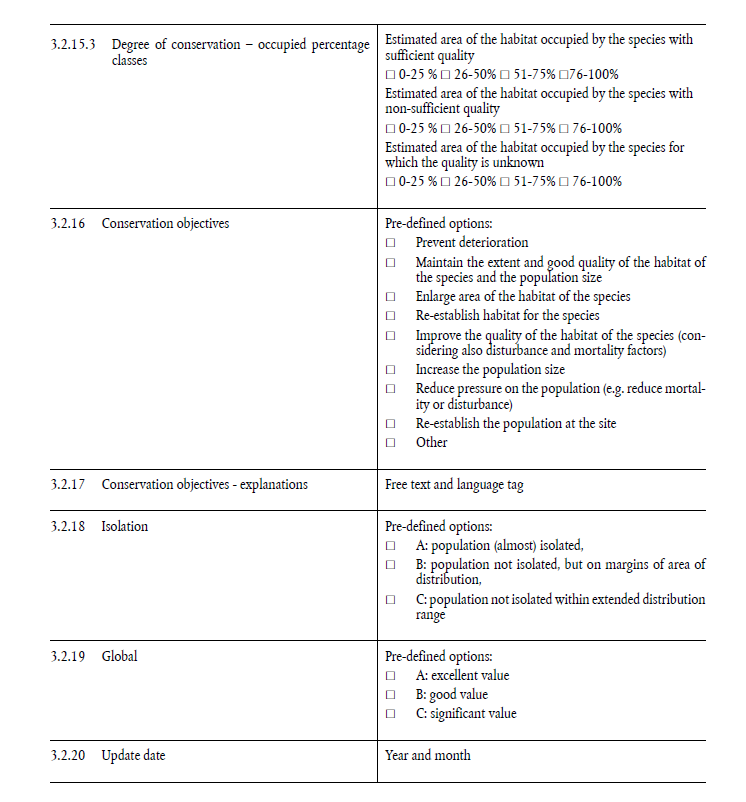


Of special interest is the POPULATION category in the SDF. This category evaluates the relative size and density of the population in the Natura 2000 area with that of the national population. Populations should be assigned to different categories (A, B, C and D) depending on the ratio of the population size and the population size in the national territory. As proposed for criterion A(b), populations should be classified in relation to these percentages:

| Significant A | : | 100 % ≥ p > 15 %, |
| --- | --- | --- |
| Significant B | : | 15 % ≥ p > 2 %, |
| Significant C | : | 2 % ≥ p > 0 %. |

In all areas where the species is observed, regardless the size of the population, the population should be considered “significant”**;** the population size will help to categorize the “significant” populations in A, B or C. Furthermore, all cases where a population of the species concerned is present on the area in question in a non-significant manner must be indicated. Only in those cases where a species is rarely observed on an area, for example a vagrant individual, the population could be categorized in a fourth category D, as a “non-significant population”. In cases where the population is categorized as ‘D: non-significant’, no other indication is required for the other evaluation criteria. The European Commission criterion recommends that only those species barely observable at the area, should be officially accepted as “non-significant”, but in practice many populations that are non-vagrant or rarely observed, are classified as “non**-**significant” based on particular criterion or expert opinion (fig. S3). This is highly relevant because conservation objectives and effective measures have to be set out or referred to in legally binding acts for only species and habitat types with “significant” presence in each Natura 2000 area.

Description of the Natura 2000 SDF and the SPECIES database

We analyzed here the Natura 2000 SPECIES database of 2020. This database contains 400177 records (i.e. populations), that correspond to more than 2900 different species and approximately 20990 Natura 2000 areas.

When analyzing the 2020 Natura 2000 SPECIES database we detected at least 0.5% erroneous values in 13 of the 18 variables of the SDF, either due to typological and completion errors, or erroneous missing values (Table S1). There is a mismatch between scientific names (SPECIESNAME) and species codes (SPECIESCODE), and scientific names are not standardized. There is a significant number of records (over 50%) that do not have population size values (LOWERBOUND and UPPERBOUND). In terms of population status (POPULATION_TYPE), more than 2% of records have typological errors and more than 2% erroneous missing values. ABUNDANCE_CATEGORY show a significant number of completion errors (≥15%) (Table S1).

| Field in SDF | Content | Typological errors (%) | Completion errors (%) | Erroneous missing values (%) |
| --- | --- | --- | --- | --- |
| COUNTRY_CODE | Country code | 0 | 0 | 0 |
| SITECODE | Natura 2000 site code | * | * | 0 |
| SPECIESNAME | Scientific name of the protected species | ** | * | 0 |
| SPECIESCODE | Code the species listed in Article 4(1) and 4(2) of the bird directive 79/409/EEC and Annex II of Council Directive 92/43/EEC | <0.5 | * | ≥0.5 |
| REF_SPGROUP | Species group from the reference ETC lookup species list | 0 | * | ≥75 |
| SPGROUP | Species group | 0 | * | <0.5 |
| SENSITIVE | States if a species is sensitive or not for its publication | 0 | * | ≥15 |
| NONPRESENCEINSITE | Information about species that no longer exist on the site | 0 | * | ≥25 |
| POPULATION_TYPE | Population status for the species | ≥2 | * | ≥2 |
| LOWERBOUND/  UPPERBOUND | Limits for the species population size | 0 | <0.5 | ≥15 |
| COUNTING_UNIT | Units of population | <0.5 | ≥2 | <0.5 |
| ABUNDANCE_CATEGORY | Species population abundance category | ≥0.5 | ≥15 | <0.5 |
| DATAQUALITY | Assessment of the quality of data provided | ≥2 | ≥2 | <0.5 |
| POPULATION | Size and density of the population of the species present on the site in relation to the populations present within national territory | <0.5 | ≥2 | <0.5 |
| CONSERVATION | Degree of conservation of the features of the habitat important for the species | ≥2 | ≥0.5 | <0.5 |
| ISOLATION | Degree of isolation of the population present on the site in relation to the natural range of the species | ≥0.5 | ≥0.5 | <0.5 |
| GLOBAL | Global assessment of the value of the site for conservation of the species concerned | ≥2 | ≥0.5 | <0.5 |
| INTRODUCTION_CANDIDATE | Species referred to in Article 4 of Directive 2009/147/EC or species listed in Annex II to Directive 92/43/EEC considered as a candidate for introduction on the site | 0 | 0 | 0 |

Table S1. Percentage of typological and completion errors in the 2020 SPECIES database from Natura 2000. “Content” explains the information recorded in the specific variable of the SDF column. * Unknown; ** Not quantified.

Of the 27 Member States of the European Union, Italy is the country with the highest number of records (106563), followed by Spain (70931), Germany (43619), France (39342) and Finland (16019) (fig S1). Most of the Natura 2000 Network areas have less than 100 recorded populations, although there is great variability in the number of populations per site (with a minimum of 1 population to a maximum of 457). The SPECIES database contains species records of seven major taxonomic groups (Amphibians, Birds, Fish, Invertebrates, Mammals, Plants and Reptiles). There is great variability in the number of records of each group among Member States of the European Union (fig S2) and Birds are the taxonomic group with the highest number of records (301813).

We also detected high variability in the number of recorded populations per species. About 95% of the species have less than 1000 populations. Only a few species have more than 4000 recorded populations, such as (*Lanius collurio*), with 4,745 records, or (*Alcedo atthis*), with 4089. Species that motivated the declaration of Natura 2000 areas but are no longer present in these areas represent 1.2% of the data (4762 records). “Permanent” populations are the most abundant population status category (POPULATION_TYPE) (125958), followed by “breeding” populations (103157), and “concentrations” (97491) being the less abundant “wintering” populations (50506). About 45% of records had an assigned population size.

We identified a large amount of data deficient (DD) populations, being by far the most abundant category in data quality assessment (162298 records reported as DD or simply marked as NA). The proportion of DD and non-DD records is highly variable between Member States of the European Union, and represents: 1) more than 75% of the data in countries such as Cyprus, Italy, and Romania; 2) between 25 and 75% in Belgium, Croatia, the Czech Republic, Denmark, France, Germany, Greece, Latvia, Lithuania, Portugal, Slovenia, and Spain and 3) less than 25% in Austria, Bulgaria, Estonia, Finland, Hungary, Ireland, Luxembourg, Malta, the Netherlands, Poland, Slovakia, and Sweden (fig. 1). We must emphasize that the number of records reported by country is also very variable.


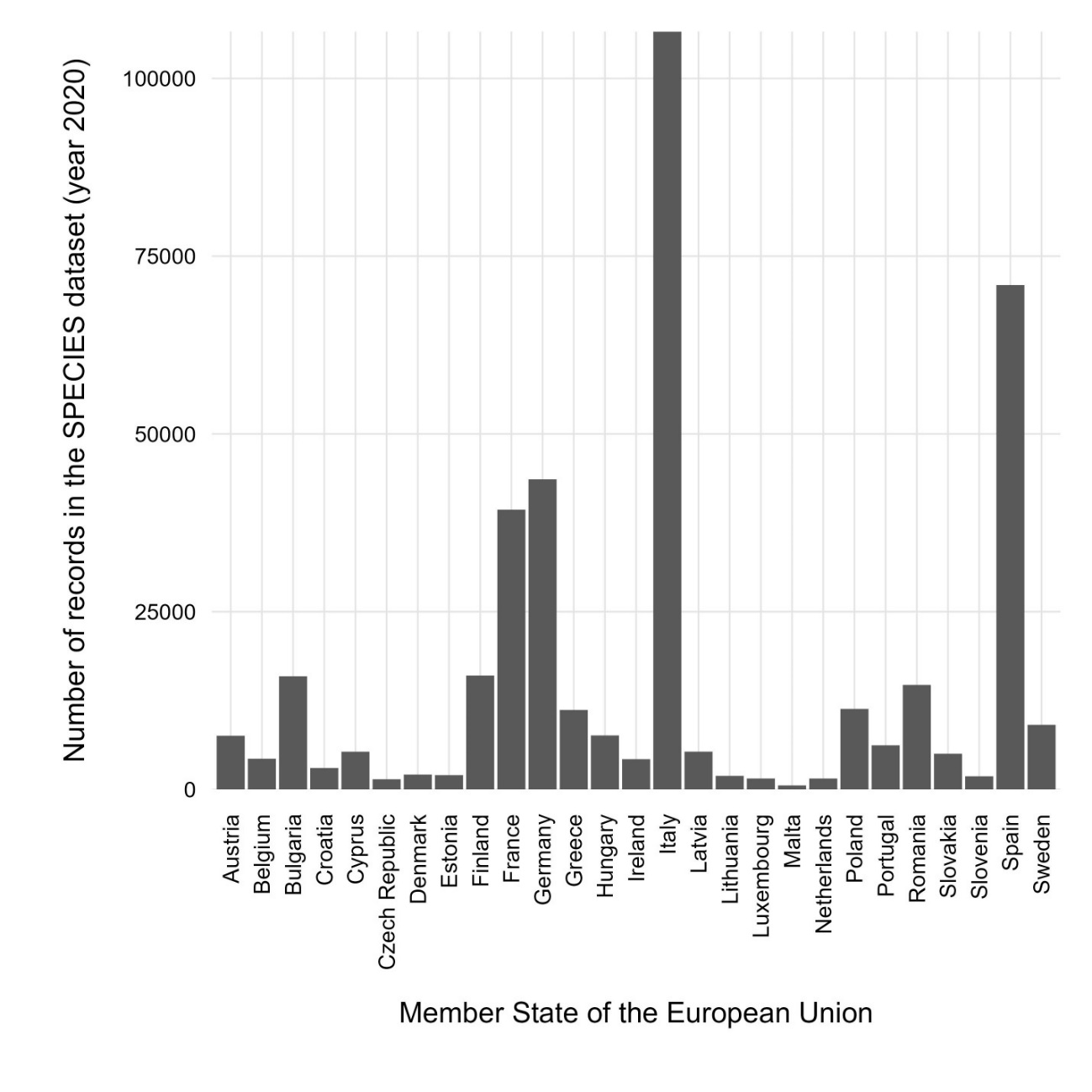


**Fig. S1. Total number of populations recorded at each Natura 2000 area by each EU State Member in 2020 database**. EU state members are shown in the X axis by alphabetical order.


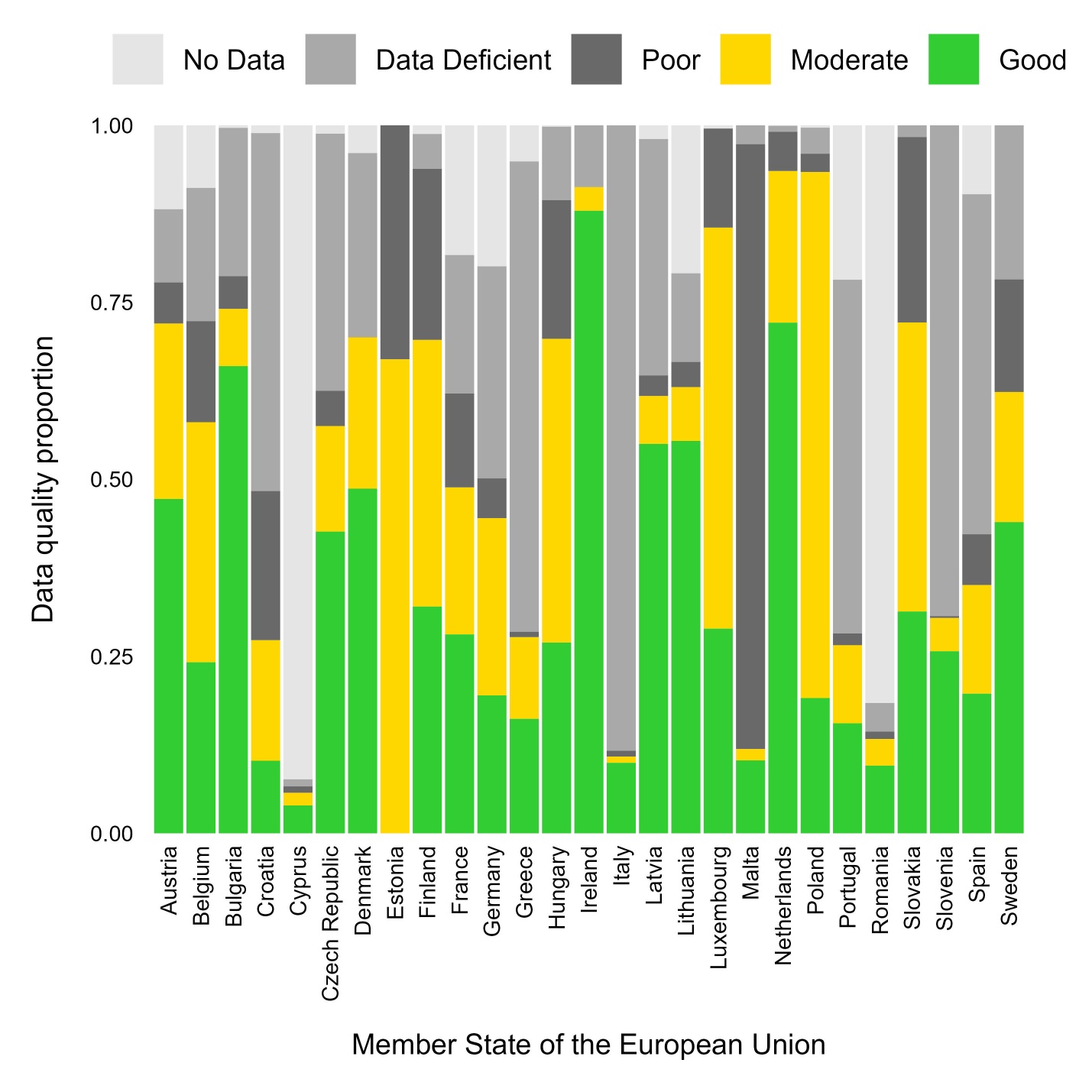


Fig. S2. Proportion of records in the 2020 Species Nature 2000 database by data quality category for each country. No data, records for which data quality is not recorded; data deficient, good, moderate, and poor are the data quality categories on the Standard Data Form.


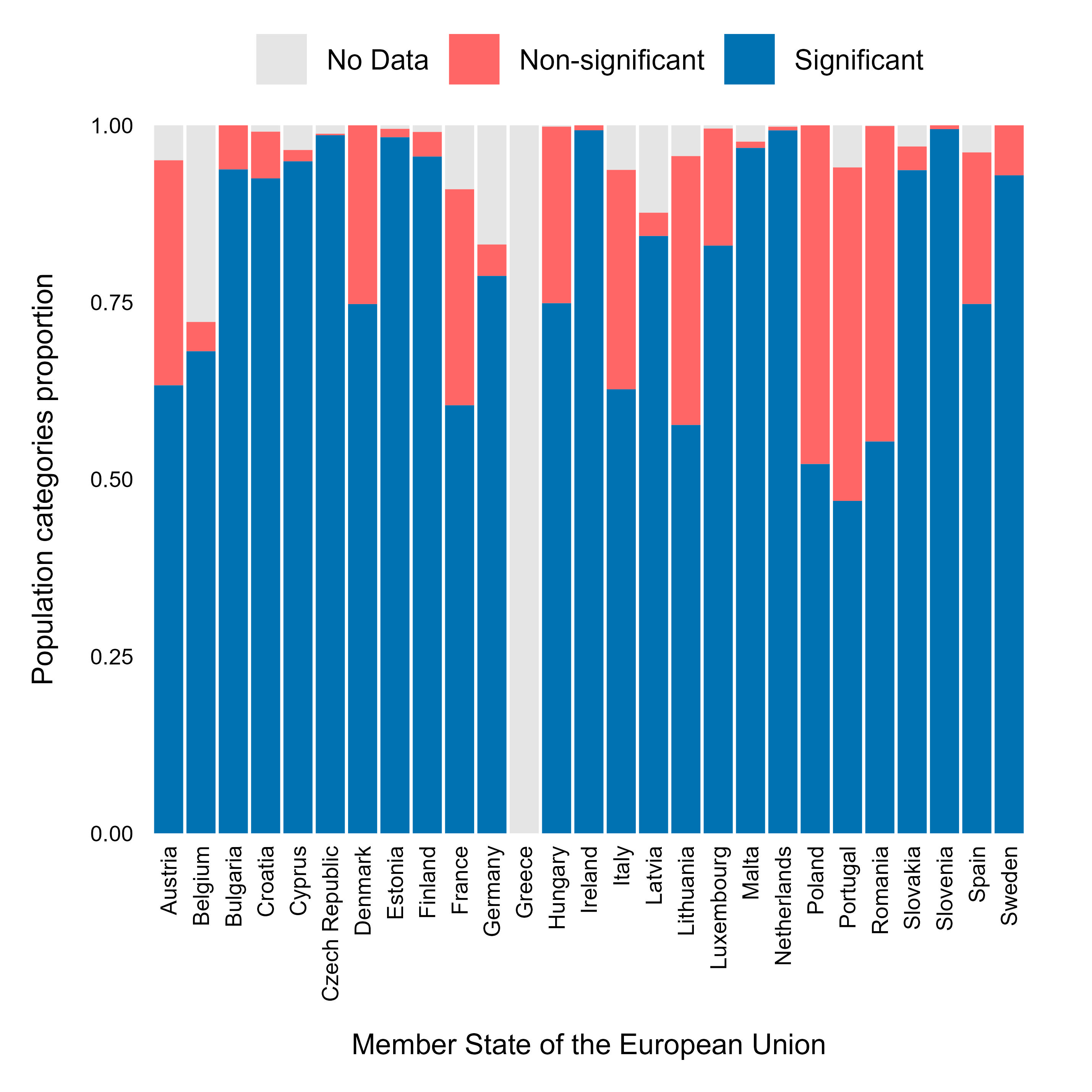


Fig. S3. Proportion of populations categorized in the Standard Data Form as not significant and significant by EU Member States (gray, proportion of populations with no information about its population category).

**A)
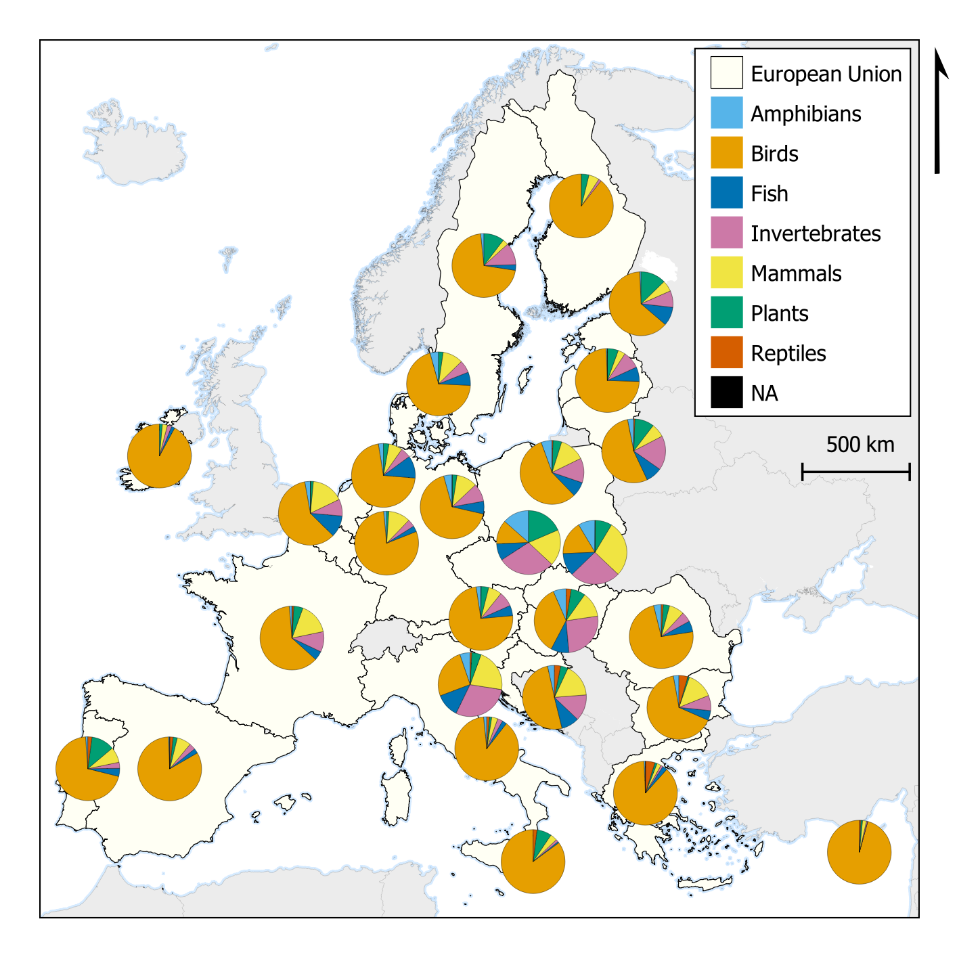
**


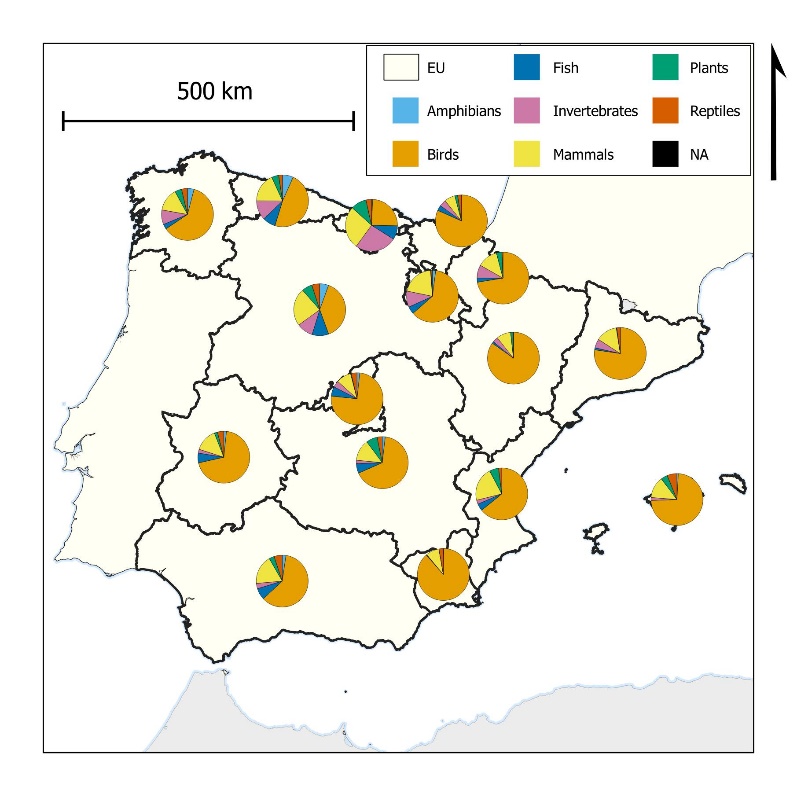


**B)**

**Fig. S4. Proportion of populations recorded by taxonomic group at Natura 2000 areas in EU State Members.** Proportion of populations recorded by taxonomic group at Natura 2000 areas among countries (A) and among regions in Spain (B). Borders between regions in Spain are shown by black lines.

a)

| **COUNTRY** | **CODE** | **Plants** | **Invertebrates** | **Fish** | **Amphibians** | **Reptiles** | **Birds** | **Mammals** | **TOTAL** |
| --- | --- | --- | --- | --- | --- | --- | --- | --- | --- |
| Austria | AT | 43 | 50 | 29 | 6 | 2 | 283 | 24 | 437 |
| Belgium | BE | 12 | 15 | 14 | 5 | 3 | 97 | 15 | 161 |
| Bulgaria | BG | 21 | 36 | 26 | 6 | 7 | 205 | 24 | 325 |
| Cyprus | CY | 22 | 3 | 1 | 0 | 13 | 256 | 17 | 312 |
| Czechia | CZ | 41 | 32 | 16 | 6 | 0 | 48 | 11 | 154 |
| Germany | DE | 30 | 40 | 29 | 3 | 1 | 307 | 15 | 425 |
| Denmark | DK | 8 | 12 | 9 | 2 | 0 | 97 | 7 | 135 |
| Estonia | EE | 18 | 18 | 6 | 2 | 0 | 116 | 4 | 164 |
| Spain | ES | 265 | 38 | 35 | 4 | 23 | 409 | 30 | 804 |
| Finland | FI | 46 | 33 | 0 | 1 | 0 | 134 | 9 | 223 |
| France | FR | 795 | 342 | 52 | 20 | 23 | 315 | 60 | 1607 |
| Greece | GR | 38 | 19 | 53 | 6 | 10 | 177 | 13 | 316 |
| Croatia | HR | 23 | 35 | 44 | 6 | 7 | 127 | 20 | 262 |
| Hungary | HU | 43 | 64 | 20 | 5 | 4 | 101 | 21 | 258 |
| Ireland | IE | 4 | 8 | 7 | 0 | 0 | 111 | 6 | 136 |
| Italy | IT | 89 | 40 | 26 | 16 | 10 | 393 | 22 | 596 |
| Lithuania | LT | 15 | 18 | 12 | 2 | 1 | 83 | 4 | 135 |
| Luxembourg | LU | 7 | 10 | 4 | 2 | 0 | 109 | 9 | 141 |
| Latvia | LV | 22 | 27 | 14 | 2 | 1 | 146 | 7 | 219 |
| Malta | MT | 11 | 4 | 1 | 0 | 2 | 165 | 3 | 186 |
| The Netherlands | NL | 5 | 11 | 10 | 2 | 0 | 97 | 8 | 133 |
| Poland | PL | 39 | 37 | 20 | 4 | 2 | 219 | 20 | 341 |
| Portugal | PT | 153 | 25 | 21 | 2 | 6 | 249 | 18 | 474 |
| Romania | RO | 47 | 53 | 33 | 6 | 6 | 310 | 28 | 483 |
| Sweden | SE | 55 | 30 | 4 | 2 | 0 | 195 | 8 | 294 |
| Slovenia | SI | 28 | 34 | 29 | 6 | 2 | 119 | 15 | 233 |
| Slovakia | SK | 48 | 45 | 27 | 5 | 1 | 123 | 22 | 271 |

b)

| **COUNTRY** | **CODE** | **Plants** | **Invertebrates** | **Fish** | **Amphibians** | **Reptiles** | **Birds** | **Mammals** |
| --- | --- | --- | --- | --- | --- | --- | --- | --- |
| Austria | AT | 0,098 | 0,114 | 0,066 | 0,014 | 0,005 | 0,648 | 0,055 |
| Belgium | BE | 0,075 | 0,093 | 0,087 | 0,031 | 0,019 | 0,602 | 0,093 |
| Bulgaria | BG | 0,065 | 0,111 | 0,080 | 0,018 | 0,022 | 0,631 | 0,074 |
| Cyprus | CY | 0,071 | 0,010 | 0,003 | 0,000 | 0,042 | 0,821 | 0,054 |
| Czechia | CZ | 0,266 | 0,208 | 0,104 | 0,039 | 0,000 | 0,312 | 0,071 |
| Germany | DE | 0,071 | 0,094 | 0,068 | 0,007 | 0,002 | 0,722 | 0,035 |
| Denmark | DK | 0,059 | 0,089 | 0,067 | 0,015 | 0,000 | 0,719 | 0,052 |
| Estonia | EE | 0,110 | 0,110 | 0,037 | 0,012 | 0,000 | 0,707 | 0,024 |
| Spain | ES | 0,330 | 0,047 | 0,044 | 0,005 | 0,029 | 0,509 | 0,037 |
| Finland | FI | 0,206 | 0,148 | 0,000 | 0,004 | 0,000 | 0,601 | 0,040 |
| France | FR | 0,495 | 0,213 | 0,032 | 0,012 | 0,014 | 0,196 | 0,037 |
| Greece | GR | 0,120 | 0,060 | 0,168 | 0,019 | 0,032 | 0,560 | 0,041 |
| Croatia | HR | 0,088 | 0,134 | 0,168 | 0,023 | 0,027 | 0,485 | 0,076 |
| Hungary | HU | 0,167 | 0,248 | 0,078 | 0,019 | 0,016 | 0,391 | 0,081 |
| Ireland | IE | 0,029 | 0,059 | 0,051 | 0,000 | 0,000 | 0,816 | 0,044 |
| Italy | IT | 0,149 | 0,067 | 0,044 | 0,027 | 0,017 | 0,659 | 0,037 |
| Lithuania | LT | 0,111 | 0,133 | 0,089 | 0,015 | 0,007 | 0,615 | 0,030 |
| Luxembourg | LU | 0,050 | 0,071 | 0,028 | 0,014 | 0,000 | 0,773 | 0,064 |
| Latvia | LV | 0,100 | 0,123 | 0,064 | 0,009 | 0,005 | 0,667 | 0,032 |
| Malta | MT | 0,059 | 0,022 | 0,005 | 0,000 | 0,011 | 0,887 | 0,016 |
| The Netherlands | NL | 0,038 | 0,083 | 0,075 | 0,015 | 0,000 | 0,729 | 0,060 |
| Poland | PL | 0,114 | 0,109 | 0,059 | 0,012 | 0,006 | 0,642 | 0,059 |
| Portugal | PT | 0,323 | 0,053 | 0,044 | 0,004 | 0,013 | 0,525 | 0,038 |
| Romania | RO | 0,097 | 0,110 | 0,068 | 0,012 | 0,012 | 0,642 | 0,058 |
| Sweden | SE | 0,187 | 0,102 | 0,014 | 0,007 | 0,000 | 0,663 | 0,027 |
| Slovenia | SI | 0,120 | 0,146 | 0,124 | 0,026 | 0,009 | 0,511 | 0,064 |
| Slovakia | SK | 0,177 | 0,166 | 0,100 | 0,018 | 0,004 | 0,454 | 0,081 |

c)

| **COUNTRY** | **CODE** | **Plants** | **Invertebrates** | **Fish** | **Amphibians** | **Reptiles** | **Birds** | **Mammals** |
| --- | --- | --- | --- | --- | --- | --- | --- | --- |
| Austria | AT | 0,04 | 0,08 | 0,05 | 0,02 | 0,00 | 0,74 | 0,07 |
| Belgium | BE | 0,01 | 0,09 | 0,11 | 0,03 | 0,00 | 0,60 | 0,16 |
| Bulgaria | BG | 0,01 | 0,08 | 0,05 | 0,03 | 0,04 | 0,66 | 0,14 |
| Cyprus | CY | 0,01 | 0,01 | 0,00 | 0,00 | 0,01 | 0,96 | 0,02 |
| Czechia | CZ | 0,19 | 0,29 | 0,08 | 0,14 | 0,00 | 0,12 | 0,18 |
| Germany | DE | 0,02 | 0,09 | 0,06 | 0,04 | 0,00 | 0,67 | 0,11 |
| Denmark | DK | 0,02 | 0,06 | 0,07 | 0,04 | 0,00 | 0,70 | 0,10 |
| Estonia | EE | 0,13 | 0,08 | 0,09 | 0,01 | 0,00 | 0,63 | 0,06 |
| Spain | ES | 0,02 | 0,03 | 0,02 | 0,01 | 0,02 | 0,83 | 0,07 |
| Finland | FI | 0,04 | 0,01 | 0,00 | 0,00 | 0,00 | 0,89 | 0,05 |
| France | FR | 0,05 | 0,10 | 0,05 | 0,01 | 0,01 | 0,62 | 0,16 |
| Greece | GR | 0,01 | 0,01 | 0,02 | 0,01 | 0,05 | 0,88 | 0,02 |
| Croatia | HR | 0,04 | 0,13 | 0,09 | 0,04 | 0,03 | 0,50 | 0,17 |
| Hungary | HU | 0,07 | 0,26 | 0,09 | 0,07 | 0,03 | 0,36 | 0,13 |
| Ireland | IE | 0,01 | 0,02 | 0,02 | 0,00 | 0,00 | 0,92 | 0,03 |
| Italy | IT | 0,01 | 0,03 | 0,02 | 0,02 | 0,01 | 0,88 | 0,03 |
| Lithuania | LT | 0,10 | 0,17 | 0,08 | 0,03 | 0,01 | 0,54 | 0,07 |
| Luxembourg | LU | 0,01 | 0,04 | 0,03 | 0,01 | 0,00 | 0,79 | 0,11 |
| Latvia | LV | 0,05 | 0,09 | 0,07 | 0,01 | 0,00 | 0,74 | 0,04 |
| Malta | MT | 0,07 | 0,01 | 0,01 | 0,00 | 0,02 | 0,85 | 0,04 |
| The Netherlands | NL | 0,03 | 0,05 | 0,11 | 0,03 | 0,00 | 0,71 | 0,07 |
| Poland | PL | 0,04 | 0,12 | 0,08 | 0,06 | 0,00 | 0,57 | 0,13 |
| Portugal | PT | 0,12 | 0,03 | 0,04 | 0,01 | 0,02 | 0,70 | 0,08 |
| Romania | RO | 0,03 | 0,05 | 0,06 | 0,04 | 0,01 | 0,74 | 0,08 |
| Sweden | SE | 0,10 | 0,11 | 0,03 | 0,02 | 0,00 | 0,71 | 0,03 |
| Slovenia | SI | 0,05 | 0,30 | 0,12 | 0,05 | 0,01 | 0,25 | 0,22 |
| Slovakia | SK | 0,08 | 0,26 | 0,12 | 0,09 | 0,00 | 0,17 | 0,28 |

Table S3. **a) Number of species b) proportion of species and c) proportion of populations registered by taxonomic group at Natura 2000 areas in each EU State Members.**

**Appendix S2**. Actual criteria to determine the “significance of populations”

With the objective of knowing the criteria used in each of the Member States to determine the “significance of populations” in the SDF, during the months of October and November 2021, we contacted the different countries and regions within countries, when relevant. The first contact was made through the Spanish Ministry for the Ecological Transition and the Demographic Challenge, and we wrote to the members of the 'Expert Group on Reporting' of both directives. Through this approach we obtained three responses. A second round was then carried out through personal contact to The National Nature & Biodiversity contact points (NCPs) provided by the European Commission and to the contacts provided on the websites of the Natura 2000 of each country. With this approach we obtained most of the answers. We did a third round of contact and show them our gathered information to get some more answers and to check if they agree with our conclusions. The responses from the different Member States are shown below by country in Table S2.

| **Region** | **Country** | **Management of the areas falls on different regional bodies in the country?** | **The data reported to the SDFs have a proportion of DD / NA records >25% for the Data Quality variable?** | **Does the SM or region explicitly recommend other criteria to determine significant populations that the one recommended by the EC?** * | **Even if not officially, does the SM or region use other criteria to determine significant populations?** | **Is expert opinion used in some step of the decision-making process?** | **When expert opinion is used?** |
| --- | --- | --- | --- | --- | --- | --- | --- |
| Burgenland | Austria (AT) | Yes | No | No | Yes | Yes | To decide if a population is not significant |
| Oberösterreich |  |  |  | No | Yes | Sometimes | To decide if a population is not significant |
| Tyrol |  |  |  | No | Yes | Yes | To decide if a population is not significant |
| Flanders | Belgium (BE) | Yes | No | No | Yes | Sometimes | To decide whether a population is not significant, what conservation measures to apply, and in what cases |
| Valonia |  |  | Yes | No | Yes | Unknown | Unknown |
|  | Bulgaria (BG)* | Unknown | No | No | No | Yes | To decide if a population is not significant |
|  | Croatia (HR) | NA | Yes | NA | NA | NA | NA |
|  | Republic of Cyprus (CY) | NA | Yes | NA | NA | NA | NA |
|  | Czech Republic (CZ) | Yes | Yes | No | Yes | Sometimes | Unknown |
|  | Denmark (DK) | NA | Yes | NA | NA | NA | NA |
|  | Estonia (EE) | NA | No | NA | NA | NA | NA |
|  | Finland (FI) | No | No | Unknown | Yes | Yes | To decide if a population is not significant |
|  | France (FR) | Unknown | Yes | No | No | Yes | To decide if a population is not significant |
|  | Germany (DE) | Yes | Yes | No | Yes | Yes | To decide if a population is not significant |
|  | Greece (EL/GR) | NA | Yes | NA | NA | NA | NA |
|  | Hungary (HU) | Unknown | No | Yes | Yes | Yes | To apply exceptions; to decide if a population is not significant |
|  | Ireland (IE) | NA | No | NA | NA | NA | NA |
|  | Italy (IT) | NA | Yes | NA | NA | NA | NA |
|  | Latvia (LV) | Unknown | Yes | No | Yes | Yes | To evaluate SDF data and critically review all other available data |
|  | Lithuania (LT) | NA | Yes | NA | NA | NA | NA |
|  | Luxembourg (LU) | NA | No | NA | NA | NA | NA |
|  | Malta (MT) | Yes | No | Yes | Yes | Yes | Unknown |
|  | Netherlands (NL)* | Unknown | No | Yes | Yes | Unknown | Unknown |
|  | Poland (PL)* | Unknown | No | No | Yes | Sometimes | To apply exceptions (to decide if a population is not significant) |
|  | Portugal (PT) | NA | Yes | NA | NA | NA | NA |
|  | Romania (RO) | NA | Yes | NA | NA | NA | NA |
|  | Slovakia (SK)* | No | No | No | Yes | Yes | To decide if a population is not significant |
|  | Slovenia (SI) | Unknown | Yes | Yes | Yes | Yes | To decide if a population is not significant (especially when there is not enough field data) |
|  | Spain (ES) | Yes | Yes | No | No | Yes | To decide if a population is not significant |
|  | Sweden (SE) | Yes | No | No | Yes | Yes | To decide if a population is not significant |

**Table S2.** Criteria for registering data in the SDF for different EU State Members (SM). The EU criteria for considering a population non-significant is: “a species is barely observable at the site, for example a vagrant species”*.* Information comes from answers from EU Natura 2000 contacts from each country. NA: means not available information. NA indicates that we could not get the information from that country. * These countries provided information on the management, decision-making and criteria used, however they did not answer on the third round to validate the table.

Appendix S3. Species occurrence and data on population size

Based on the previous exploration of the SPECIES database, and with the objective to assess the consequences of the disparities in criteria and dedicated effort among countries, we mapped the occurrence and available data on population size of several species.

We first mapped the occurrence and data on population size of the most common and widely distributed terrestrial birds in Natura 2000: the European turtle dove (*Streptopelia turtur*) (fig.1), and the red-backed shrike (*Lanius collurio*), the European nightjar (*Caprimulgus europaeus*), the common cuckoo (*Cuculus canorus*), the black kite (*Milvus migrans*), and the common kingfisher (*Alcedo atthis*) (fig. S5)*.* We found evidence that there is a heterogeneity in the sampling effort and/or in the data reported by the different Member States or even regions within countries. We observed a large number of areas without a recorded presence of these species, even if they are distributed throughout the European geography (fig. S5).

We also mapped the occurrence and population size of other common and widely distributed European species of other non-Bird groups based on recorded data on Natura 2000 areas. Specifically, we studied the distribution of two invertebrates, the Marsh Fritillary (*Euphydryas aurinia*) (fig S6) in the IUCN list under Least concern category, and the European stag beetle (*Lucanus cervus*) (fig S7)*;* two amphibians, the great crested newt (*Triturus cristatus*) and the Yellow-bellied toad (*Bombina variegata*) (fig S8), both in the IUCN list under Least concern category. We found many areas with recorded presence of the species but no information on population size, while those with some information on populations size were a minority, and, in many cases, aggregated nationally or regionally (e.g.: in Catalonia, Germany, and Bulgaria) (figs. S6-S8).

The consequences of the disparities in dedicated effort and allocation of resources across Nature 2000, and in criteria used to identify significant populations among countries are non-negligible and heterogeneities translate in a dataset that do not allow assessment or comparison of the conservation status of species.

A)


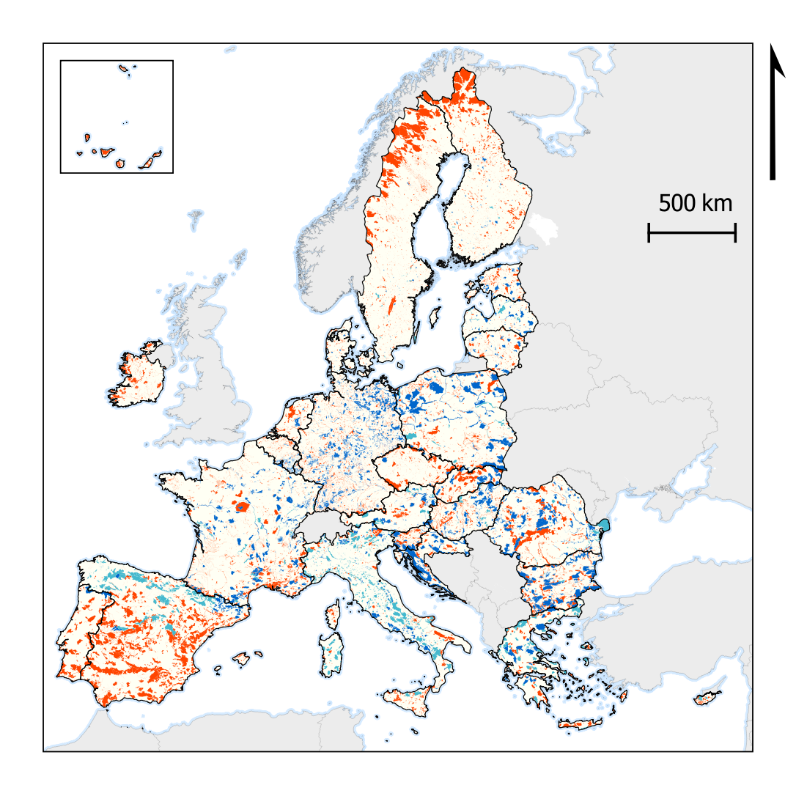

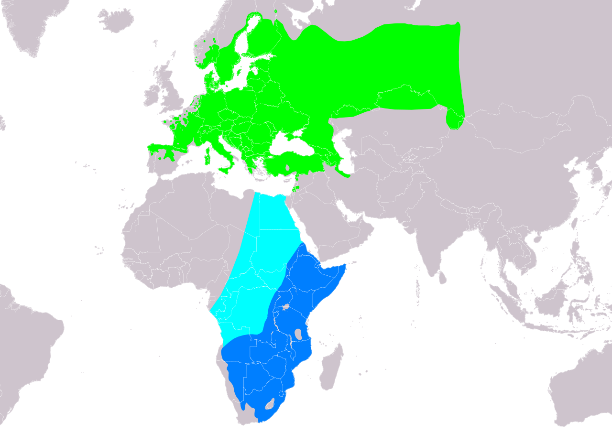


B)


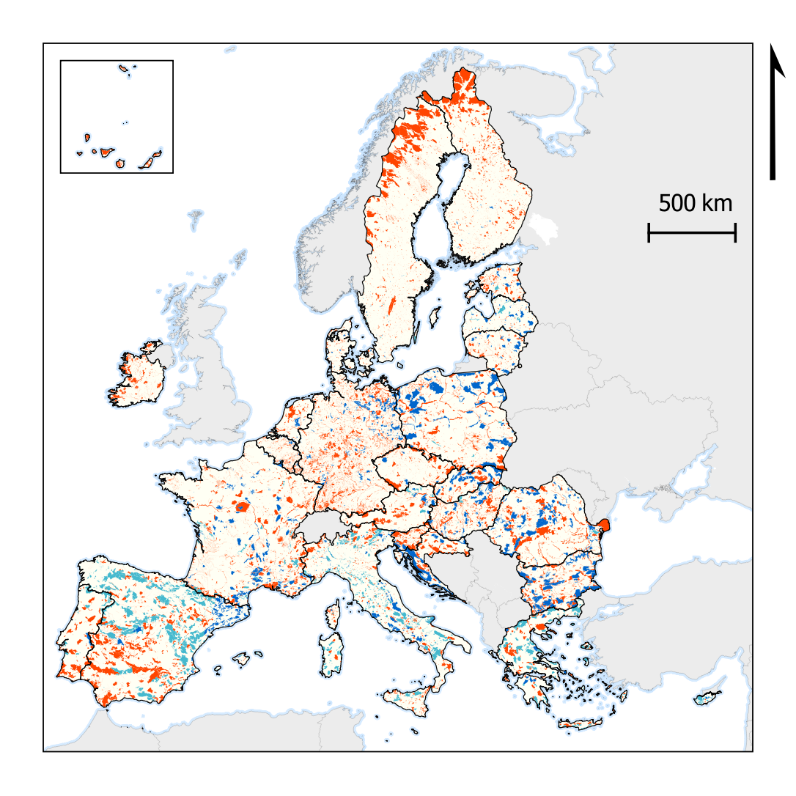

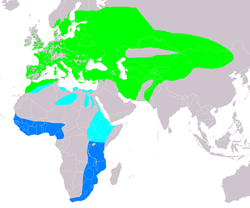


C)


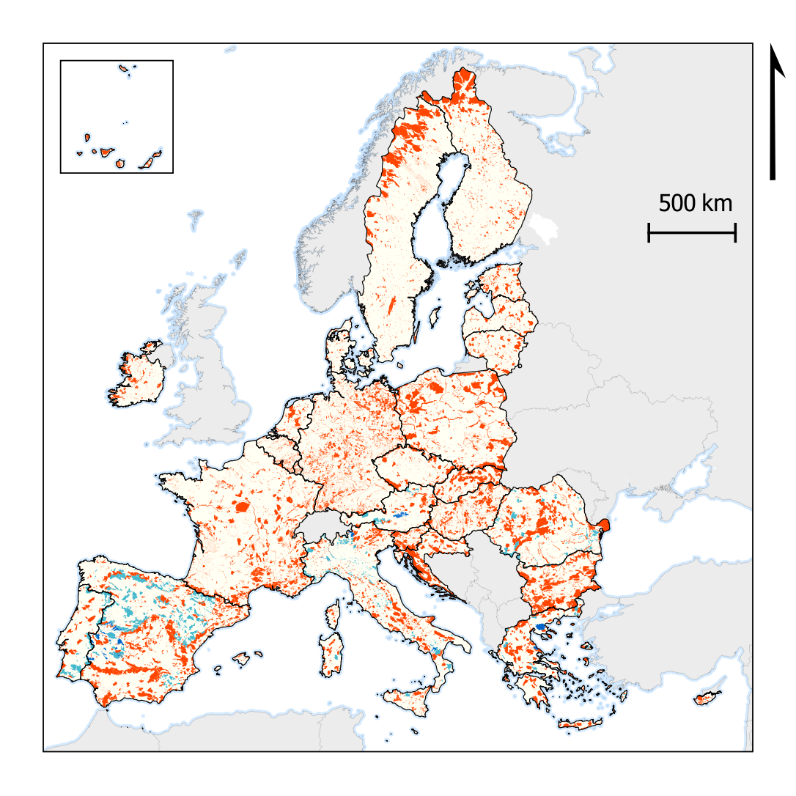

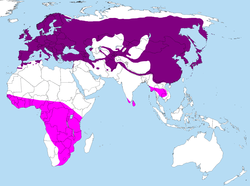


D)


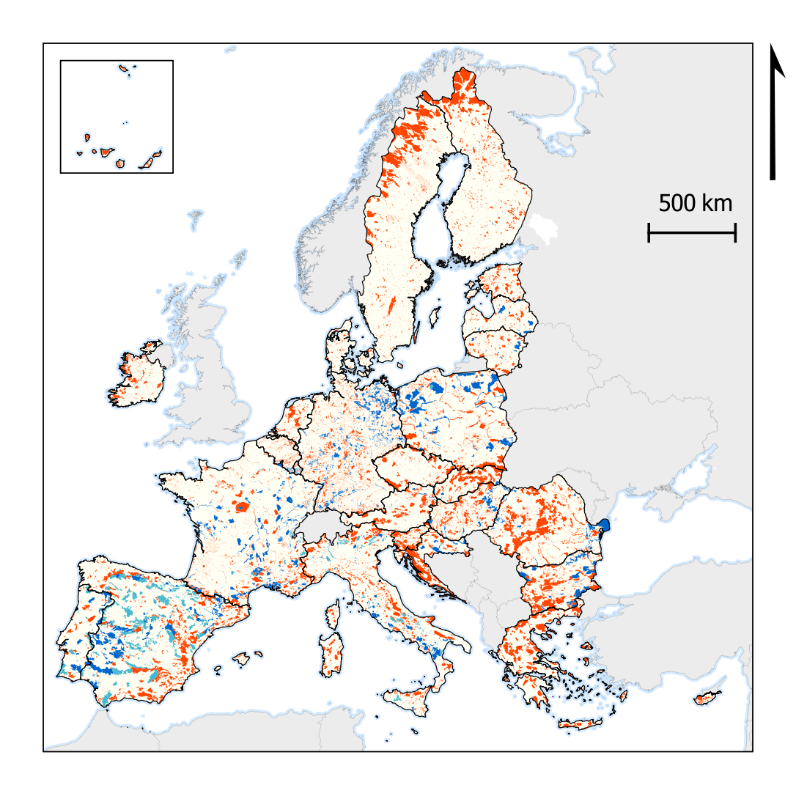

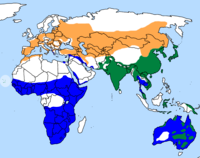


E)

**Fig. S5.** **The map of distribution** **of the most common and widely distributed European terrestrial birds in Natura 2000**: In each left panel, occurrence and population size from the 2020 version of the Natura 2000 global database are shown. Light blue: 2000 Natura areas with recorded presence of the species but no information on population size; dark blue: 2000 Natura areas with recorded presence of the species, also with information on population size; red: 2000 Natura areas with no recorded presence of the species. In some cases, national or regional frontiers are visible. (A) the red-backed shrike *Lanius collurio*, (B) the European nightjar *Caprimulgus europaeus*, (C) the common cuckoo *Cuculus canorus*, (D) the black kite *Milvus migrans*, and (E) the common kingfisher *Alcedo atthis*. We also show in the right panel the known species distribution. Red-backed shrike distribution from <https://es.wikipedia.org/wiki/Lanius_collurio>; in green breeding area, in light blue, transit area and in dark blue, wintering area. European nightjar distribution from <https://es.wikipedia.org/wiki/Caprimulgus_europaeus>; in green breeding area, in light blue, migration area and in dark blue, wintering area. Common cuckoo distribution from [https://es.wikipedia.org/wiki/Cuculus_canorus;](https://es.wikipedia.org/wiki/Cuculus_canorus;%20)  in purple breeding area and in pink wintering area. Black kite distribution from <https://es.wikipedia.org/wiki/Milvus_migrans>; in green resident area, in orange breeding area and in blue wintering area. Common kingfisher distribution from <https://es.wikipedia.org/wiki/Alcedo_atthis>; in green resident area, in yellow breeding area and in blue wintering area.


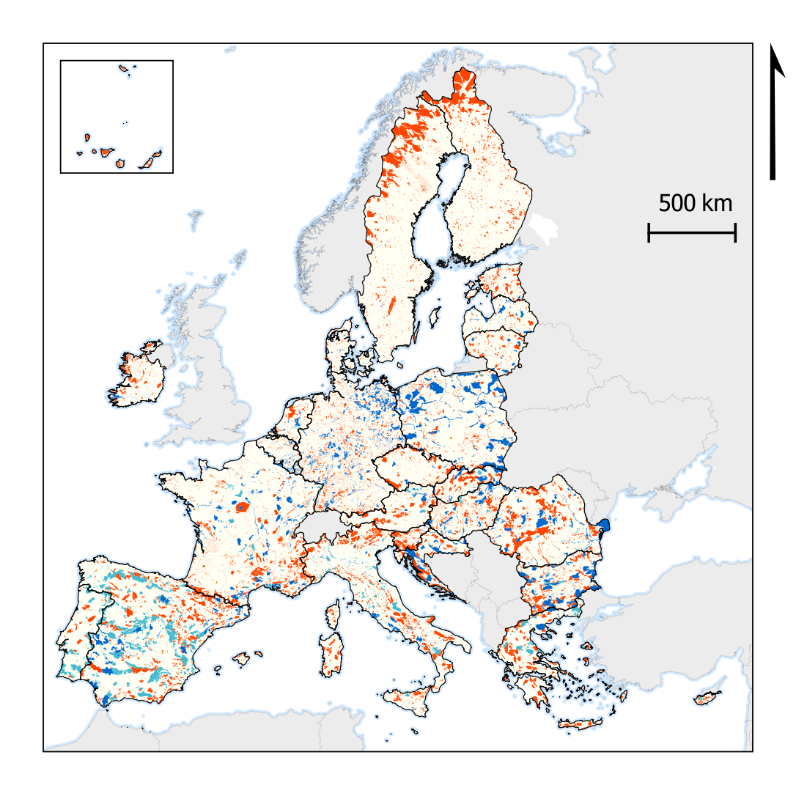

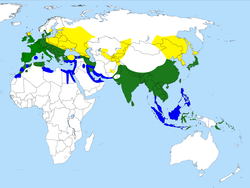


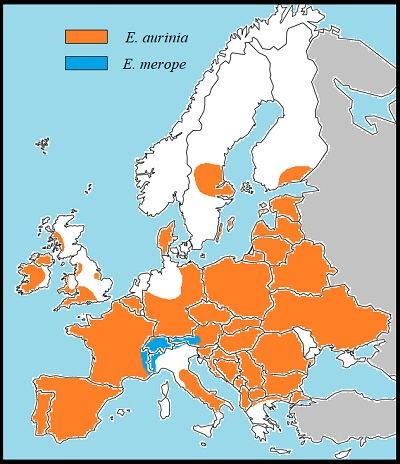
**
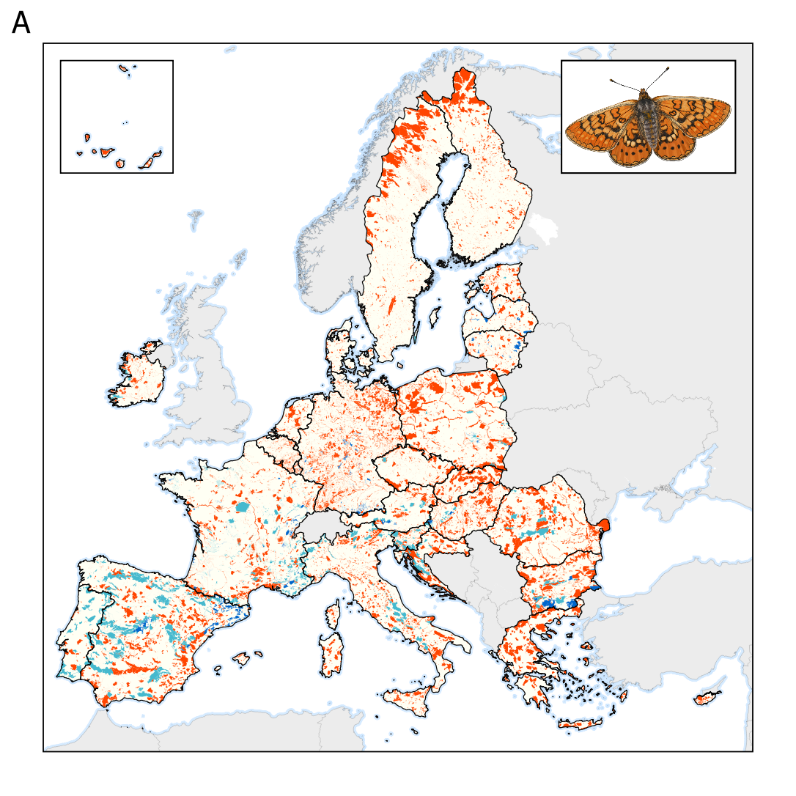
**

**Fig. S6. Map of distribution of the butterfly Marsh Fritillary *Euphydryas aurinia****.* In the left panel we show in light blue: 2000 Natura areas with recorded presence of the species but no information on population size; dark blue: 2000 Natura areas with recorded presence of the species, also with information on population size; red: 2000 Natura areas with no recorded presence of the species. We also show in the right panel, in orange, the known species distribution (<https://www.guypadfield.com/marshfritillary.html> ).


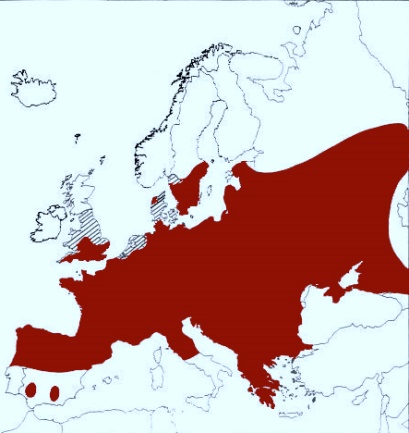

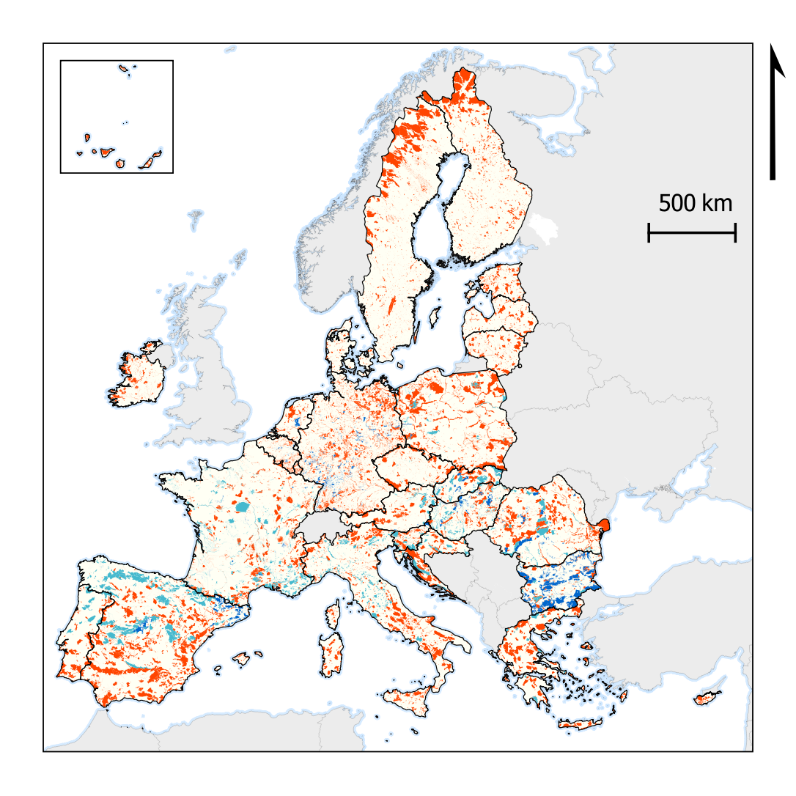


**Fig. S7**. **Occurrence and population size data available of one beetle *Lucanus cervus* in Natura 2000 Database**. In the left panel we show in light blue: 2000 Natura areas with recorded presence of the species but no information on population size; dark blue: 2000 Natura areas with recorded presence of the species, also with information on population size; red: 2000 Natura areas with no recorded presence of the species. We also show in the right panel the known species distribution from <https://en.wikipedia.org/wiki/Lucanus_cervus>.

A)


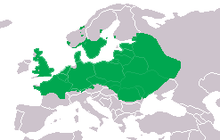

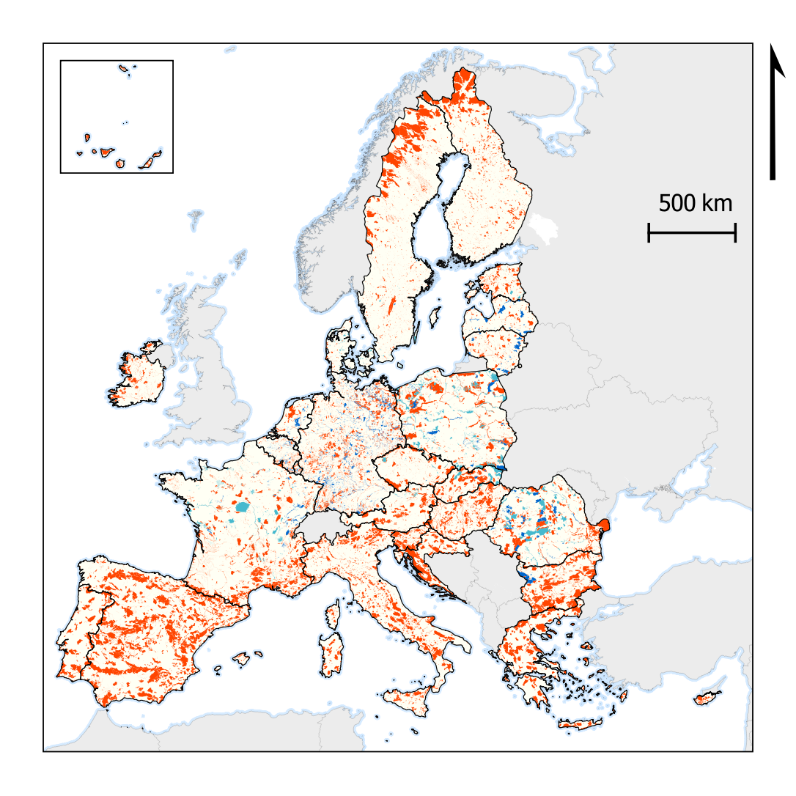


B)


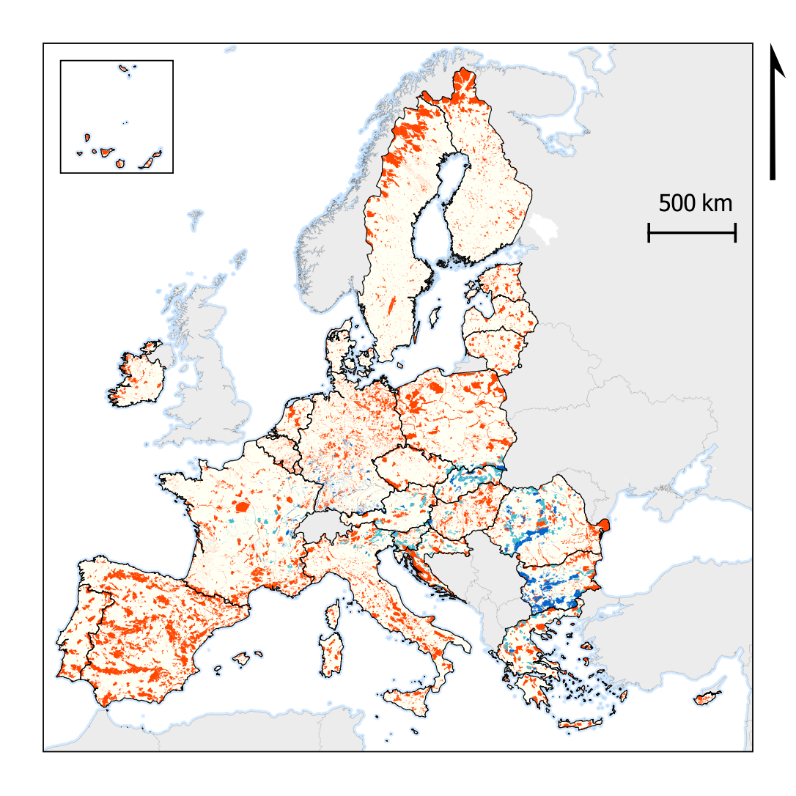

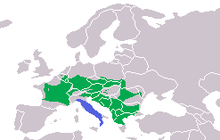


**Figure S8**. **Occurrence and population size data available of two amphibian species in Natura 2000 Database.** In each left panel we show in light blue: 2000 Natura areas with recorded presence of the species but no information on population size; dark blue: 2000 Natura areas with recorded presence of the species, also with information on population size; red: 2000 Natura areas with no recorded presence of the species. A) *Triturus cristatus* with 2313 populations in Natura 2000 and the category of threat Least Concern and B) *Bombina variegata*, with 1570 populations in Natura 2000 and the category of threat Least Concern. We also show in the right panel in green, the known species distribution from <https://en.wikipedia.org/wiki/Northern_crested_newt> and <https://en.wikipedia.org/wiki/Yellow-bellied_toad>.
